# Fitness cost of resistance for lumefantrine and piperaquine-resistant *Plasmodium berghei* in a mouse model

**DOI:** 10.1186/s12936-015-0550-5

**Published:** 2015-01-28

**Authors:** Winnie R Gimode, Daniel M Kiboi, Francis T Kimani, Hannah N Wamakima, Marion W Burugu, Francis W Muregi

**Affiliations:** Department of Biochemistry and Biotechnology, Kenyatta University, P.O. Box 43844, Nairobi, Kenya; Department of Biochemistry, Jomo Kenyatta University of Agriculture and Technology, P.O. Box 62 000, Nairobi, Kenya; Centre for Biotechnology Research and Development, Kenya Medical Research Institute (KEMRI), P.O. Box 54840, Nairobi, Kenya; Centre for Traditional Medicine and Drug Research, Kenya Medical Research Institute (KEMRI), P.O. Box 54840 Nairobi, Kenya; Directorate of Research and Development, Mount Kenya University, P.O. Box 342–01000, Thika, Kenya

**Keywords:** Fitness cost, Drug resistance, Piperaquine, Lumefantrine, Malaria

## Abstract

**Background:**

The evolution of drug-resistant parasites is a major hindrance to malaria control, and thus understanding the behaviour of drug-resistant mutants is of clinical relevance. The study aimed to investigate how resistance against lumefantrine (LU) and piperaquine (PQ), anti-malarials used as partner drugs in artemisinin-based combination therapy (ACT), impacts parasite fitness. This is important since resistance to ACT, the first-line anti-malarial regimen is increasingly being reported.

**Methods:**

The stability of *Plasmodium berghei* ANKA strain that was previously selected for LU and PQ resistance was evaluated using the 4-day assay and established infection test in mice. Fitness cost of resistance was determined by comparing parasites proliferation rates in absence of drug pressure for the drug-exposed parasites between day 4 and 7 post-infection (pi), relative to the wild-type. Statistical analysis of data to compare mean parasitaemia and growth rates of respective parasite lines was carried out using student’s *t*-test and one-way analysis of variance, with significance level set at p<0.05.

**Results:**

During serial passaging in the absence of the drug, the PQ-resistant parasite maintained low growth rates at day 7 pi (mean parasitaemia, 5.6% ± 2.3) relative to the wild-type (28.4% ± 6.6), translating into a fitness cost of resistance of 80.3%. Whilst resistance phenotype for PQ was stable, that of LU was transient since after several serial passages in the absence of drug, the LU-exposed line assumed the growth patterns of the wild-type.

**Conclusions:**

The contrasting behaviour of PQ- and LU-resistance phenotypes support similar findings which indicate that even for drugs within the same chemical class, resistance-conferred traits may vary on how they influence parasite fitness and virulence. Resistance-mediating polymorphisms have been associated with less fit malaria parasites. In the absence of drug pressure in the field, it is therefore likely that the wild-type parasite will out-compete the mutant form. This implies the possibility of reintroducing a drug previously lost to resistance, after a period of suspended use. Considering the recent reports of high failure rates associated with ACT, high fitness cost of resistance to PQ is therefore of clinical relevance as the drug is a partner in ACT.

## Background

Most of the global malaria burden is due to *Plasmodium falciparum* and, therefore, many efforts for prevention and eradication of malaria have focused on this parasite [1]. Anti-malarial drug resistance poses a very significant threat in the fight against malaria, making adequate management of this infectious disease increasingly difficult [2,3] and thus resulting in high mortality rates. Development of resistance in malaria is through selection of variant parasites that are favoured under conditions of drug pressure [4]. Resistance becomes a clinical problem when the frequency of the resistant variant threatens the effectiveness of empirical drug therapy. The level of exposure of the parasite population to the drug influences the selection of resistant variants [5,6]. Artemisinin and its derivatives are currently deemed the most potent anti-malarials [7]. They are presently administered in combination with at least one other long-acting anti-malarial, such as lumefantrine (LU), to prolong their lifespan by reducing the emergence of drug-resistant parasites [8]. Currently used artemisinin-based combination therapy (ACT) include artemether-lumefantrine (ALU, Coartem®), dihydroartemisinin-piperaquine (DHA-PQ, Artekin®), artesunate-mefloquine, artesunate-sulfadoxine-pyrimethamine, artesunate-amodiaquine and artesunate-pyronaridine [9].

Lumefantrine (LU) and piperaquine (PQ) are both long-acting anti-malarial drugs currently used as part of ACT. The terminal elimination half-life of a drug is an important determinant of the propensity for an anti-malarial drug to select for resistance. Therefore, the mismatch between the short-acting artemisinin derivative and the long-acting partner drug provides selection pressure for emergence of resistant parasites, since one drug is rapidly eliminated and the other drug persists alone. This exposes reinfections to sub-therapeutic level of the slowly-eliminated drug and may be the starting point for development of tolerance/resistance towards the long-acting partner [10,11]. The half-life of LU is about 3–5 days [12]. Emerging reports indicate that the use of LU (aryl amino alcohol) in ALU selects for parasites that are less susceptible to this drug combination [13-15]. Polymorphisms in PfMDR1, particularly the variant N86, and amplification of the encoding gene (*pfmdr1*) have been associated with reduced susceptibility to LU in Africa and Asia [16,17]. Additionally, parasites with the wild type copy of PfCRT show reduced susceptibility to LU, as indicated by both field studies and in vitro assays [18]. Piperaquine (bis-4-aminoquinoline) possesses a prolonged half-life of up to 5 weeks. It has the longest terminal elimination half-life of the available artemisinin partner drugs [19-21]. Consequently, it is rare for new infections to occur after treatment with dihydroartemisinin-piperaquine DHA-PQ as compared to using other artemisinin combinations [22-25]. The long half-life of PQ however may also expose new infections to sub-optimal drug levels [26] because of its slow elimination, hence selecting for PQ-resistant parasites.

Studies performed using rodent malaria models to determine the magnitude of selection pressures have given ambivalent findings. For example, Rosario et al. [27,28] found a slight advantage of resistance, while Chawira et al. [29] reported rapid loss of resistance once drug selection was removed. If drug resistance does indeed incur a cost on parasite growth rate in the absence of drugs, it is expected that the virulence and transmissibility of resistant parasites would also be reduced. This is because parasite growth rate and virulence are positively associated with the parasite’s transmission rate, and hence its Darwinian fitness [30].

In this study, lines of the ANKA strain of *Plasmodium berghei* that had been previously selected for resistance against LU and PQ were revived and inoculated into mice. The study was initiated to establish the impact of resistance on the fitness of LU- and PQ-resistant parasites, in the absence of the drugs. It would be essential to know the behavior of these parasites, as the two drugs are used as partner drugs in ACT. The definition of fitness includes an organism’s ability to survive, reproduce and be transmitted [31]. The fitness cost of resistance, therefore, is the reduced ability of the resistant population to grow and multiply, with respect to the ancestor population, in the absence of the selection pressure (in this case anti-malarials) [32,33]. Studies have confirmed that a reduction in drug use would benefit the fitter susceptible (without resistance-associated mutations) strains, enabling them to out-compete resistant strains over time [34-38]. Experimental studies of the costs imposed by resistance are usually aimed at comparing the survival and multiplication rates between sensitive and resistant parasites, usually in pair wise competition experiments [35]. Establishing the relations between resistance and fitness requires experiments on isogenic strains [39]. In a study assessing the fitness cost paralleled with the benefit of resistance of a *P. falciparum* drug resistance mutation on parasite growth in vitro, results indicated that mutated parasites grew less in low drug concentrations due to a predominating fitness cost [40,41]. Moreover, studies on the rodent malaria parasites *P. berghei* and *Plasmodium chabaudi* also show that while allowing the resistant parasite to evade the deleterious effects of the drug, mutations compromise their natural physiological functions [42,43].

It is important to recognize that there are many types of cost-free and costly forms of parasite resistance. If fitness costs are nevertheless observed in laboratory experiments, it is probable that there will also be clinical conditions under which the resistance would impose a fitness burden [44].

## Methods

### Parasites, hosts and drugs

Lumefantrine and piperaquine-resistant lines of *P. berghei*-ANKA were used for this study. The parasites, maintained in a frozen state (−80°C) at Centre for Traditional Medicine and Drug Research (CTMDR) in Kenya Medical Research Institute (KEMRI) had been subjected to selective LU and PQ pressure up to the 73^rd^ and 64^th^ passages respectively [45]. Wild-type isogenic strains of *P. berghei* parasites were revived and independent inoculation was done intraperitoneally (ip) into specific-pathogen-free (SPF) donor mice. The donor mice were then used for passaging into groups of experimental male Swiss albino mice which were 6–8 weeks old weighing 20 ± 2 g that were random-bred at KEMRI, Nairobi, Kenya, where the study was conducted. These mice had not been subjected to any previous experimental procedures. The inoculated mice were then randomized into groups of five that were housed in standard plastic cages (layered with woodchip bedding) per group and maintained in the animal facility. The animals were fed on commercial rodent pellets and water ad libitum. Bleeding of donor mice was through the cardiac puncture on anaethesia with sodium pentobarbital and blood collected into heparinized tubes. All mice that were either cured or were deemed to have completed their intended use were euthanized with sodium pentobarbital in the course of experiments.

The drugs of study, namely LU and PQ were gifts from Universal Corporation Limited and CTMDR of KEMRI, respectively. On the day of administration, the drug was freshly prepared by dissolving it in a vehicle consisting of 70% Tween-80 (*d* = 1.08 g/ml) and 30% ethanol (*d* = 0.81 g/ml) and subsequently diluted 10-fold with double distilled water (to result in a stock solution of 7% Tween-80 and 3% ethanol concentration).

### Infection of experimental mice

Parasitaemia from donor mice was adjusted downwards using phosphate saline glucose (PSG) buffer which contained 392 g/L disodium hydrogen phosphate (Na_2_HPO_4_), 0.312 g/L sodium dihydrogen phosphate (NaH_2_PO_4_), 1.7 g/L sodium chloride (NaCl) and 10 g/L glucose dissolved using double distilled water. This solution was then sterilized by autoclaving at 121°C for 5 minutes then stored at 4°C. Each of the male albino mice was infected ip with blood containing approximately 0.5% parasitized red blood cells (PRBC) in 0.1 ml inoculum. Infection was confirmed by microscopic estimation of percentage parasitaemia (%P) of the blood. The day of infection was denoted as day 0 post-infection (pi), and all experiments were done using these revived parasites to ensure isogenicity of the parasite. The inoculated mice were then randomized into groups of five. The mice were then housed in plastic cages per group and maintained in the animal facility where they were monitored twice daily in the course of study. All mice that were either cured or were deemed to have completed their intended use were euthanized with sodium pentobarbital solution (150 mg/kg body weight) in the course of experiments.

### Confirmation of resistance after a period of dormancy

To confirm resistance after cryopreservation, the drug-exposed and the wild-type revived parasites were subjected to drug sensitivity studies in a 4-day suppressive test (4DT) [46] albeit with minor variations [47]. This involved inoculation of 25 mice; 5 mice per group for every dose (total of four different doses) and one group being the control. The infection was done ip with infected erythrocytes, on day zero (D_0_). The mice were then treated for 4 consecutive days, at drug doses of 12.5, 25, 50 and 100 mg per kg body weight for the respective groups. Drug was administered orally (po) at 4 hrs, 24 hrs, 48 hrs and 72 hrs pi. Thin blood films were prepared from tail snips on day four (D_4_) post infection, and microscopically examined under immersion oil at × 1000. Every experiment set was done in parallel for the drug-exposed and drug sensitive parasites (wild-type), with monitoring being done twice daily. Sodium pentobarbital was used to euthanize mice that had been cured of malaria after treatment, and no recrudescent parasites observed by day 30 post-infection.

Parasitized cells in 6 fields were counted to give a mean parasitized cell percentage and percentage parasitaemia (%P) was calculated as; {Number of parasitized erythrocytes/(Total number of RBC per field) × 100} [48].

### Resistance stability study and serial passage

Parasites were examined for the stability of their drug response by weekly passage through mice without drug pressure [49]. LU-exposed and its wild type isogenic strain parasites were serially passaged for 22 generations that lasted over 154 days, while PQ-exposed and the respective wild-type parasite lines were passaged for 18 generations (126 days). These parasites were also sub-passaged into further groups of mice and subjected to in vivo drug sensitivity studies (with a pre-determined dose) to test for their responses to these drugs (such that for each strain, there would be both a treated group and a corresponding untreated group). Cumulative doses of 30 mg/kg (10 mg/kg/day) LU and 15 mg/kg (5 mg/kg/day) PQ, administered po over three days from day 4 pi were used, with daily monitoring of the experimental mice. This treatment regime had earlier been shown to clear all susceptible parasites, without any sign of recrudescence whatsoever. Stable resistance was defined as the maintenance of the resistance phenotype all through the passages in the absence of drug-selection pressure [47,50]. For each drug, at least two independent experiments were conducted. Drug efficacy was determined by percentage (%) parasitaemia suppression on day 7 pi after observation of Giemsa-stained thin blood smears under microscope, relative to the untreated controls. The mice survival rate (%) and drug curative effect to mice (%) relative to the untreated controls were also used as a measure of effect of these drugs on the infected mice. Mice that showed parasites on day 4 pi, but were aparasitaemic (in this case, up to 10 fields were microscopically examined and no infected erythrocytes detected) on subsequent days post-treatment up to day 30 pi were considered cured [51]. Percentage (%) parasitaemia suppression for the drugs was calculated as: 100 − {(mean parasitaemia treated/mean parasitaemia control) × 100} [52].

### Fitness cost of resistance

The parasites used in this study were isogenic (a sensitive wild-type clone and a resistant mutant derived from it) differing only on their susceptibility to the drug. Considering their isogenicity, their growth rate was used as a measure of fitness [4,43,53]. Parasite growth rate between day 4 and 7 pi in the course of serial passaging in the absence of drug was used to assess the parasites proliferation rates for the LU and PQ-exposed parasites relative to their wild-type counterpart, and thus their fitness. The percentage loss of fitness of the mutant parasites relative to the wild-type was expressed as: 100 – {(mean parasitaemia mutant/mean parasitaemia wild-type) × 100} [54]. Percentage parasitaemia was recorded in Microsoft Excel® 2010 and expressed as the mean ± standard error of mean. ED_50_ (Effective dose that reduces parasitaemia by 50%) was determined using linear regression equation in Excel, while statistical analysis of data to compare mean parasitaemia and growth rates of the appropriate parasite lines was carried out using student’s *t*-test and one-way analysis of variance (ANOVA). Statistical significance was defined for an overall error at 0.05 level (95% confidence interval).

### Ethics statement

Permission to use mice was granted by the Animal Care and Use Committee (ACUC), KEMRI, where all animal experiments were conducted after the research protocol approval by Mount Kenya University Ethics Research Committee (Permit Number: *MKU/ERC/0004/2013*) which is accredited by the National Council for Science, Technology and Innovation (NACOSTI). All efforts were made to minimize animal suffering, and animals were euthanized using 150 mg/kg body weight sodium pentobarbital solution injected intraperitoneally after their intended use. The euthanized mice were autoclaved in biohazard disposable bags at 121°C for 15 minutes before incineration to destroy all infectious agents and to avoid environmental contamination.

## Results

### Lumefantrine and piperaquine-resistance was not lost with dormancy

**Figure 1:**
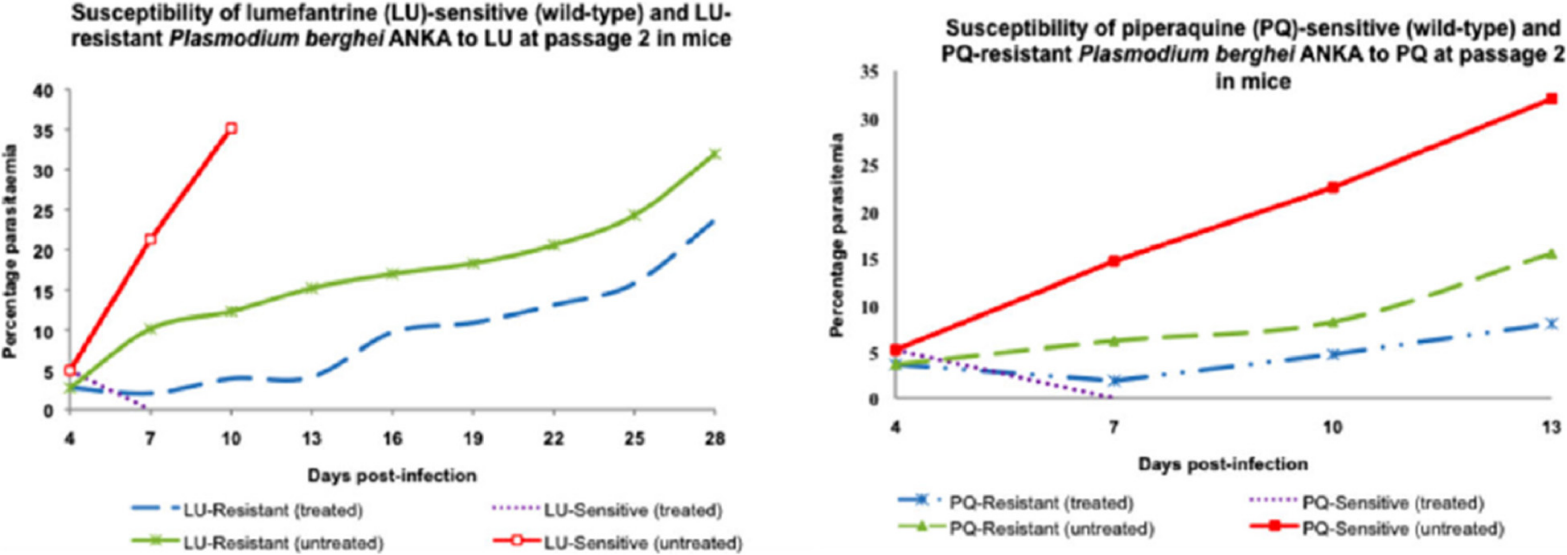
**Established infection drug sensitivity tests for lumefantrine (LU)- and piperaquine (PQ)-exposed parasites at passage 2 in mice.** Treatment was done using a dose of 10 mg/kg LU and 5 mg/kg PQ respectively, administered orally over 3 days consecutively starting from day 4 post-infection.

Parasitaemia patterns obtained after mice infected with lumefantrine (LU) or piperaquine (PQ) resistant lines were orally treated with either LU or PQ indicated that the resistance phenotype for both had not been lost with dormancy, as shown in Figure 1. Upon revival of the parasites in mice, initial drug susceptibility tests using the 4DT (4-day suppressive test) and established infection test confirmed high levels of resistance, indicating that these drug-exposed parasites did lose their resistance with dormancy.

### Lumefantrine resistance is unstable

Lumefantrine resistance phenotype was evident over the first 16 passages in the absence of drug pressure, since mice infected with LU-resistant parasites and treated with LU continued show blood stage malaria parasites which not only persisted but also grew with greater robustness, compared to the sensitive strain. Moreover, these parasites were highly resistant as a dose of up to 100 mg/kg only slightly reduced the parasitaemia whereas 5 mg/kg dose cleared the wild strain in an established infection study. Most of the untreated control mice infected with LU-exposed and wild-type parasite lines died by day 8 pi with increasing parasitaemia.

**Figure 2:**
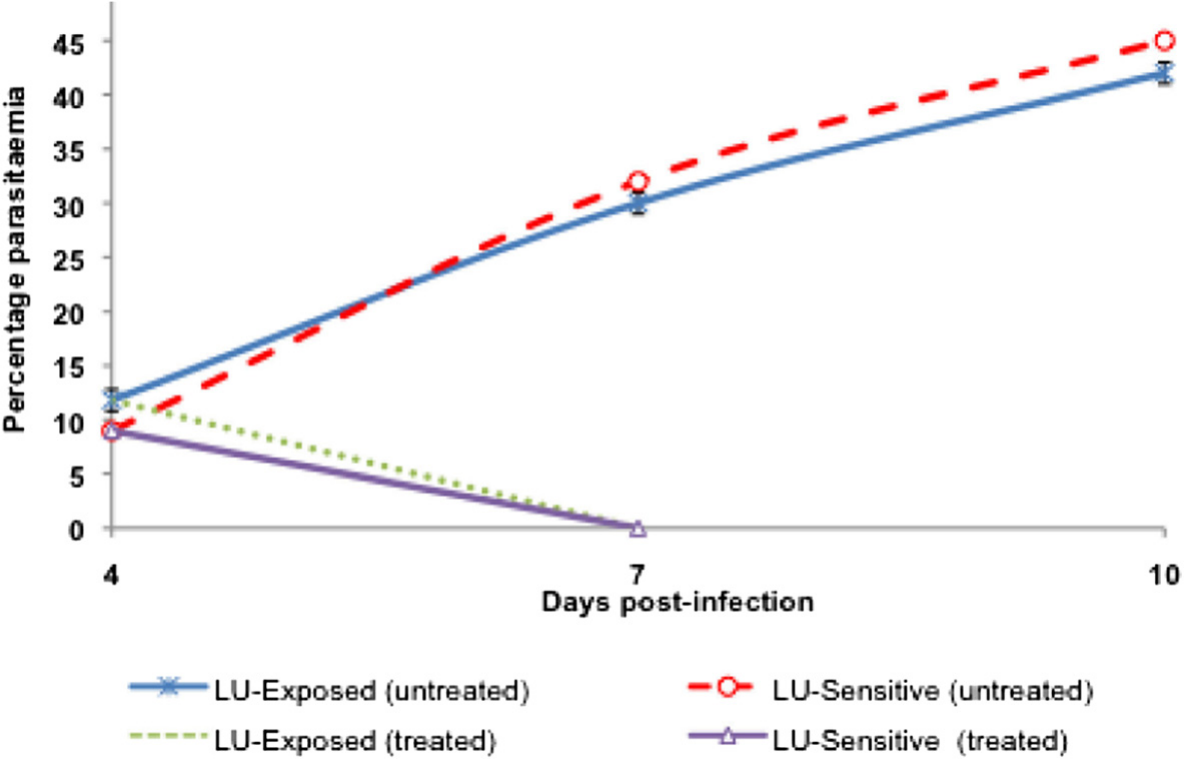
**Sensitivity of lumefantrine (LU)-exposed** ***Plasmodium berghei*** **ANKA to LU at passage 17.** A dose of 10 mg/kg/day cleared LU-exposed parasites by D_7_ post-infection (pi), confirming loss of resistance. No recrudescence was observed even by D_30_ pi.

Interestingly, from passages 17–22, the LU-treated mice infected with either the LU-exposed or wild-type parasites showed similar susceptibility to LU, where no parasites could be observed under the microscope at days 7 and 10 pi, and without any recrudescent parasites even by D_30_ pi (Figure 2). Effective doses that reduced parasitemia by 50% (ED_50_) values were useful in estimating the level of resistance of the drug-exposed parasites and were assessed at the beginning and end of study, as shown in Table 1. To determine the ED_50_, two independent experiments were performed and mean parasitaemia of mice per group was calculated, and used to estimate percentage chemo-suppression, relative to the untreated controls. A dose–response curve was then plotted and using a linear regression line (Microsoft Excel® 2010), the drug concentrations at which 50% of suppression of parasitaemia was achieved determined. At the beginning of the study (passage 2), the ED_50_ was determined as 93.31 mg/kg. However, at passage 17, there was complete loss of LU-resistance since the ED_50_ value of 1.83 mg/kg was obtained, which implies that in the course of serial passaging, these parasites may have lost resistance. This ED_50_ value was maintained over the next drug free passages (up to passage 22). Moreover, the observation that LU-exposed line assumed the patterns of its wild-type counterpart overtime indicates instability of this resistance.

**Table 1:** **Response of drug-exposed** ***Plasmodium berghei*** **ANKA strain to piperaquine and lumefantrine**

**Drugs used to treat mice infected with lumefantrine- and piperaquine-exposed** ***P. berghei*** **ANKA**						
	**Lumenfantrine (LU)**			**Piperaquine (PQ)**		
	***Parent strain***	***LU-exposed strain at passage 2***	***LU-exposed strain at passage 22***	***Parent strain***	***PQ-exposed strain at passage 2***	***PQ-exposed strain at passage 18***
**ED**_**50**_^**b**^ **(mg/kg)**	1.67^a^	93.31	1.83	1.30	85.97	81.06
**I**_**50**_^**c**^	1.00	55.87	1.09	1.00	66.13	62.35

Results are presented as effective doses that reduce parasitaemia by 50% (ED_50_) and as 50% indices of resistance (I_50_).

Drug responses (50% effective doses, ED_50s_) as well the respective 50% indices of resistance (I_50s_) of lumefantrine (LU)- and piperaquine (PQ)-exposed *Plasmodium berghei* ANKA in mice at initial stages of passaging (passage 2) and at the end of passaging (passage 22 and 18 for LU-exposed and PQ-exposed respectively).

^a^The ED_50_ of the parent strain from which both LU- and PQ-exposed strains were generated is included for comparison. ^b^Data are presented as effective doses that reduced parasitemia by 50% (ED_50_). ^c^Indices of resistance (I_50,_ defined as the ratio of the ED_50_ of the resistant line to that of the parent strain) confirm that artificially-induced PQ-resistance is stable.

### Piperaquine resistance is stable

**Figure 3:**
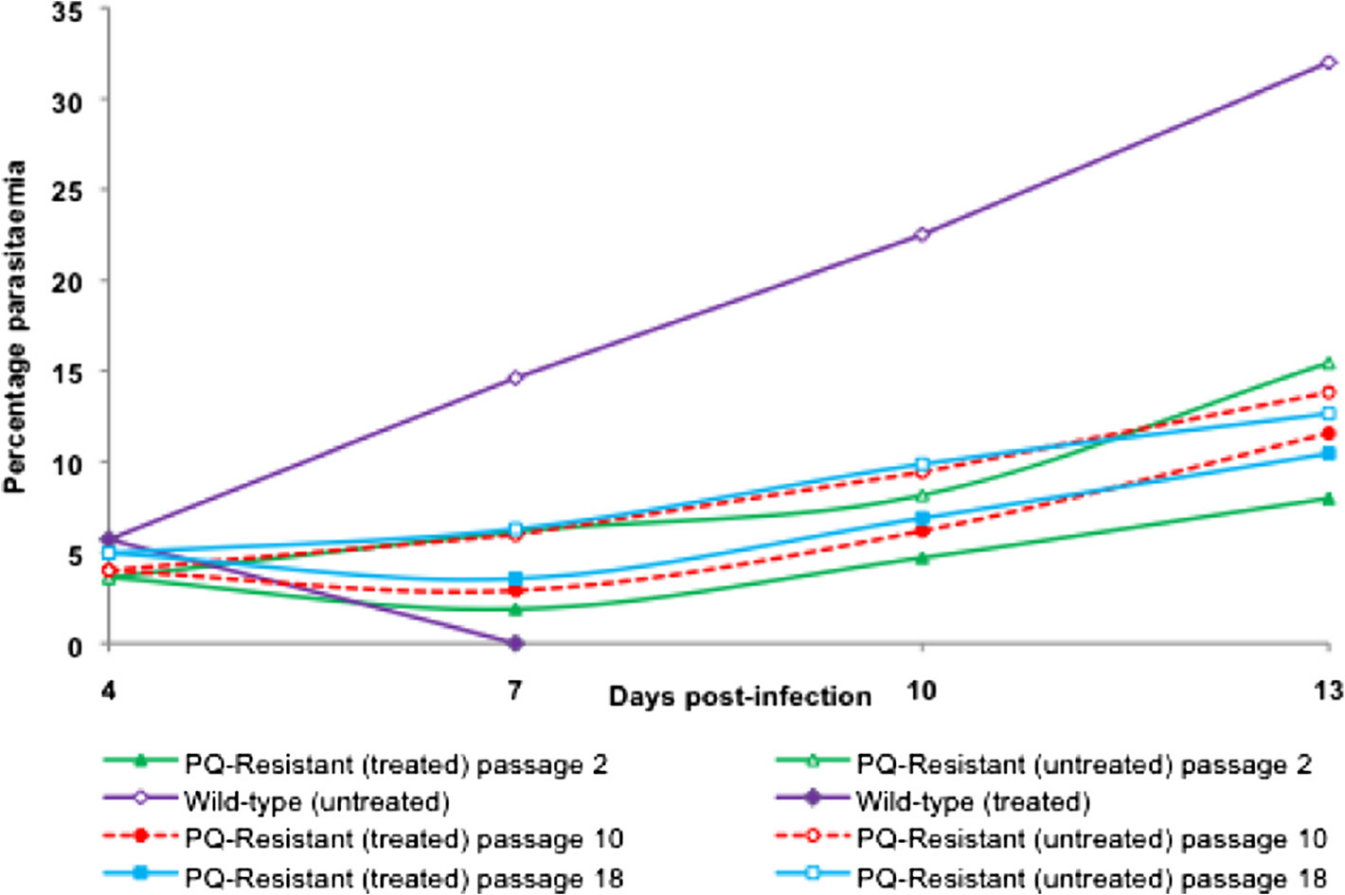
**Parasitaemia patterns for wild-type and piperaquine (PQ)-resistant** ***Plasmodium berghei*** **ANKA parasites treated with PQ at passages 2, 10 and 18.** The mice were treated once daily for 3 days with PQ (15 mg/kg cumulative dose) at every two passages. The patterns mirror each other (2, 10 and 18), confirming that the PQ-resistance is stable. No recrudescent parasites were observed following treatment of wild type infected mice, up to D_30_ post-infection.

Mice infected with wild-type parasites and treated with PQ showed complete susceptibility to PQ, since no parasites could be observed under the microscope at days 7 and 10 pi, without any recrudescent parasites appearing even by D_30_ pi. Mice infected with PQ-resistant parasite and treated with PQ showed similar (P > 0.05) patterns at passages 2 (beginning of study), 10 and 18 (end of study) in which only a slight non-significant (P > 0.05) decline in parasitaemia at D_7_ pi was observed relative to D_4_ pi, followed by gradual increase in parasitaemia on subsequent days. The ED_50_ at the 18^th^ passage was 81.02 mg/kg bw which was comparable to that at the beginning of the study (85.97 mg/kg, P > 0.05), and thus high indices of resistance (I_50_ values of 66.13 and 66.35 at passage 2 and 18, respectively) (Table 1), confirming that PQ-resistance was stable. Figure 3 shows the drug sensitivity patterns for PQ-exposed parasites at passages 2, 10 and 18.

### Lumefantrine resistance does not impose a fitness cost to the parasite

**Figure 4:**
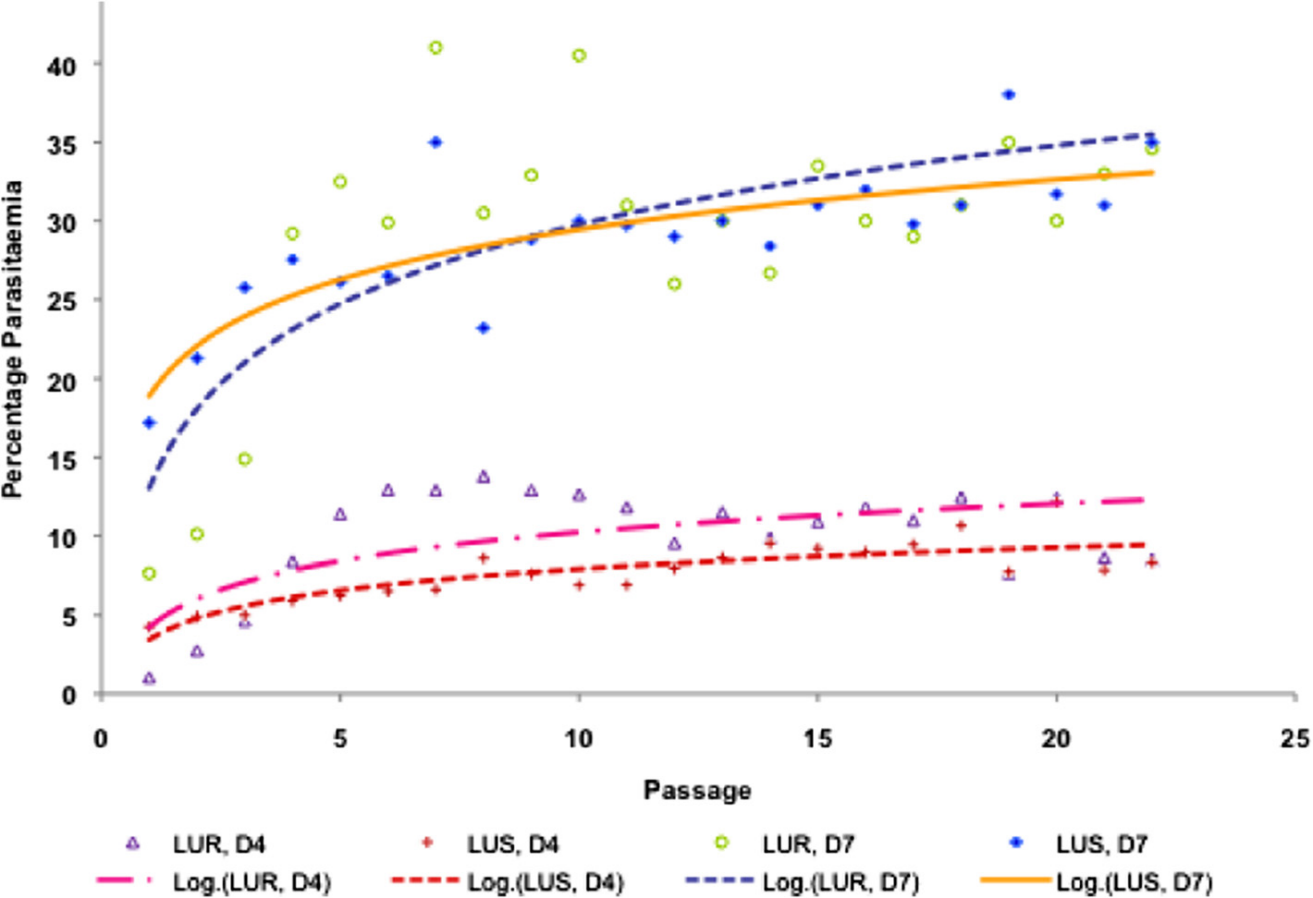
**Growth patterns for wild-type (LUS) and lumefantrine-exposed (LUR)** ***Plasmodium berghei*** **ANKA parasites between D**_4_ **and D**_7_ **post-infection (pi) in the absence of drug.** The parasitaemias were assessed at days 4 and 7 pi in the course of serial passaging of the respective parasite lines in mice for up to 22 serial passages in the absence of drug. The LUR mean parasitaemia at D4 pi was slightly higher than that of LUS, though there was no significant difference in percentage parasitaemia by D_7_ pi.

Comparison of parasitaemia at days 4 and 7 pi between LU-exposed and wild-type parasites lines in mice over 22 passages in the absence of drug revealed that previous exposure of the parasite to LU had influenced its characteristics. In the stability study, it was observed that LU-exposed parasite was growing at a different rate compared to LU-sensitive (wild-type) parasite. The LU-exposed line showed slightly lower growth rates between passages 1–3 (mean parasitaemia of 2.8 ± 1.5) relative to the wild-type parasite (mean parasitaemia of 4.7 ± 0.3), albeit not significantly different (P > 0.05). Interestingly, for the next serial passages, the LU-exposed parasite line had significantly (P = 0.01) higher parasitaemia than the wild-type parasite, implying that the resistant parasite assumed a faster growth rate from passage 4 onwards. Its mean parasitaemia of 9.98% ± 3.4 (over the 22 passages) at D4 pi was significantly different (P < 0.05) from the mean parasitaemia of LUS (7.72% ±1.9), indicating a faster growth rate than the latter at day 4pi. At passage 17 however, the parasitaemia patterns for the two strains were not significantly different (P > 0.05) and descended to the levels of the wild type, maintaining an almost similar growth pattern for the subsequent passages. During early passages, mice infected with LU-exposed parasite had longer survival relative to the wild-type parasite infected mice. In later passages however, their survival of up to D_10_ pi was similar to that of mice infected with LU-sensitive parasites. Figure 4 shows different trends observed in the parasitaemia patterns for wild-type and LU-exposed parasites.

### Piperaquine resistance imposes a fitness cost on the resistant line

**Figure 5:**
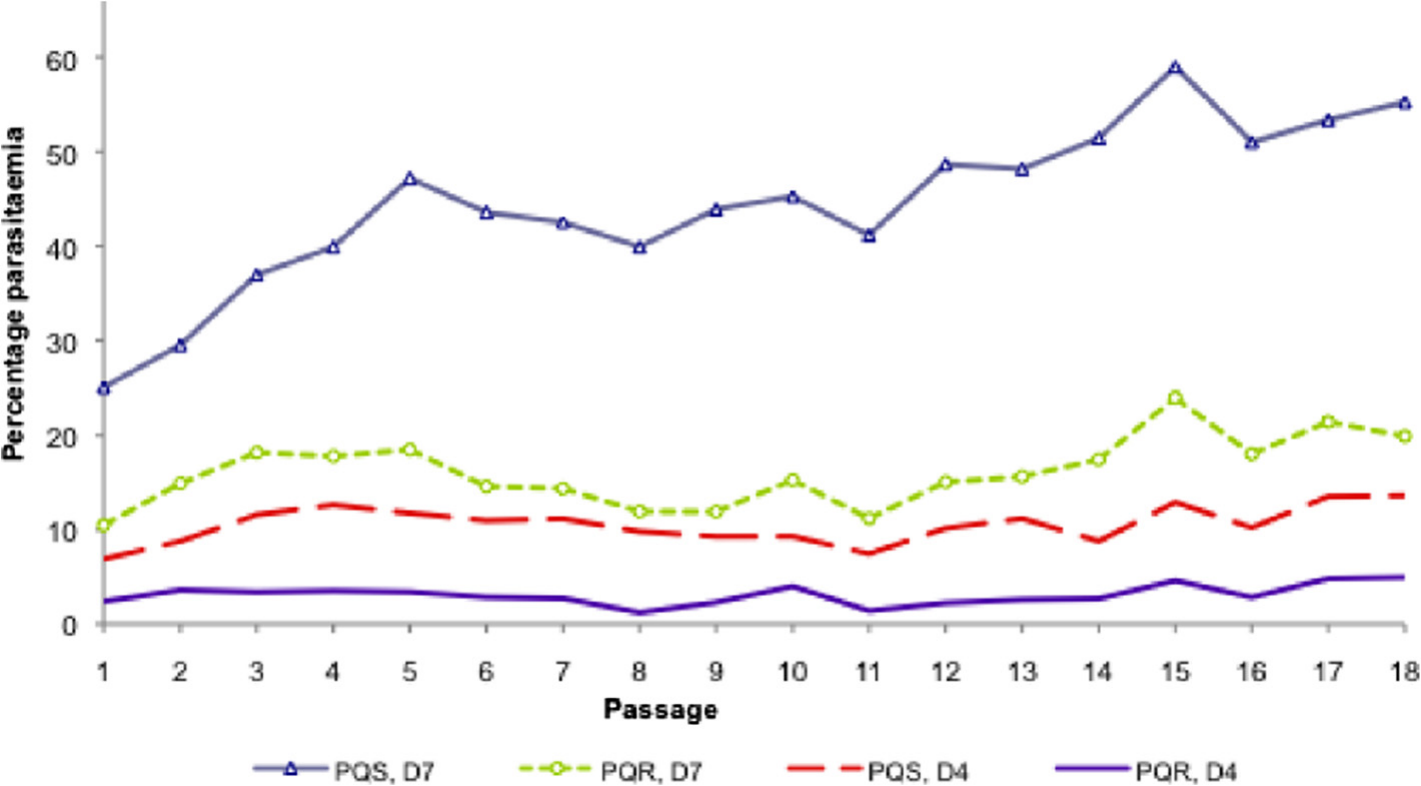
**Growth patterns for wild-type and piperaquine-resistant** ***Plasmodium berghei*** **ANKA parasites between D**_4_ **and D**_7_ **post-infection (pi) in the absence of drug.** The parasitaemias were assessed at days 4 and 7 pi in the course of serial passaging of the respective parasite lines in mice for up to 18 serial passages in the absence of drug. The mean parasitaemias for the two parasite lines were different even at day 4 pi. At day 7 pi, the wild-type parasite had five times higher parasitaemia than the PQ-resistant parasite. It was also observed that untreated mice infected with the PQ-resistant parasite had slightly longer survival (>25 days) which was statistically significant (P < 0.05) relative to the wild-type and the LU-exposed parasite-infected mice with survival of up to 10 days. All the animals succumbed to malaria infection in the absence of treatment, with the mice infected with sensitive parasites (wild-type, PQS) dying by day 10 post-infection and those infected with resistant parasites (PQR) dying by day 28 post-infection.

Parallel serial passaging of PQ-resistant and –sensitive parasite lines showed that the resistant parasite had a much slower growth rate compared to the sensitive line as shown in Figure 5. The PQR strain showed significantly low (P < 0.05) growth rates over the 18 passages (mean parasitaemia = 5.6 ± 2.3) relative to the wild-type parasite (28.4 ± 6.6) at D_7_ pi. These differences in parasitaemia translate into a five-fold faster growth rate for the wild-type parasite by D_7_ pi, and an overall asexual stage resistance cost of fitness of 80.3% for the PQ-resistant line.

## Discussion

Alterations that render an organism resistant to a drug are likely to be associated with a loss of fitness. To further the knowledge on the impact of resistance on parasite fitness, rodent malaria parasites that had been previously subjected to selective drug pressure using LU (aminoalcohol-fluorene derivative) and PQ (4-aminoquinoline), two long-acting anti-malarials currently used in combination with artemisinin derivatives, were investigated. Drug sensitivity tests on revived LU and PQ-exposed parasites confirmed that resistance was not lost even after cryopreservation for over four years. Some studies have revealed that parasites can revert to the sensitive phenotype after cryopreservation [55]. For example, a study by Hunt et al. [55] confirmed reduced susceptibility to artemisinin, but the phenotype was not stable. Deviations in resistance stability and fitness costs of resistance may occur due to differences in mechanisms of resistance for different drugs. Other mechanisms of sustaining resistance in these parasites could have also been involved, including transient changes in gene expression, which may account for the loss of resistance after cryopreservation of parasites observed by Hunt and colleagues. The fact that these parasites revived and had high indices of resistance (I_50,_ defined as the ratio of the ED_50_ of the resistant line to that of the parent strain) confirmed the stability of this artificially-induced resistance.

When both LU- and PQ-exposed parasite lines were serially passaged in the absence of the drug, it was observed that the effect of drug-exposure impacted their growth and multiplication capacities differently. In this study, results point out that at the initial phases (passages 1–3) of serial passaging, the LU exposed parasites multiplied at a slower rate. However, this trend changed from passage 4 when the growth rate of LUR was much faster, having the mice die by day 10 at passage 5 and beyond, (with increasing parasitaemia), whereas initially they could survive even up to Day 28 (passage 1). The differences observed between LU-exposed parasites and their wild-type isogenic strain could have been due to epigenetic modification. These include the heritable changes in gene function that occur without a change in the nucleotide sequence [56]. Epigenetic factors include DNA methylations, histone modifications, and microRNAs, and may explain how cells with identical DNA can differentiate into diverse cell types with different phenotypes [57]. Epigenetic effects may have been involved in that; the previous continuous exposure of the parasites to LU might have triggered epigenetic adaptations. These probably increased the parasites’ tolerance to the drug, but were manifested only in a transient manner and were consequently lost after stopping drug treatment and during passaging. The data suggests that resistance to LU seems to have no negative impact on the growth of these parasites, in the absence of drug pressure.

It is probable that for LU, the prevalent strains may have lost the mutant gene(s), or unrelated, susceptible strains may have coincidentally replaced the former strains [31]. Theoretically, the latter is expected to be much more common, since the accumulation of compensatory mutations is one of the barriers to genetic reversions [31,58]. Resistance might have been due to genetic mutations that were not sustainable by the parasites and therefore lost in the absence of treatment, as confirmed at passage 17. Since a fixed dose (10 mg/kg) was in use during the sensitivity tests, it is feasible that loss of resistance might have occurred gradually and was only noted when this dose cleared even the LU-exposed parasites. This is because supplementary studies at the initial passaging phases showed that even a dose of up to 100 mg/kg for 3 days could only suppress but not clear these parasites. This was moreover emphasized by the fact that the ED_50_ at the beginning of the study for the LUR parasites was found to be 93.31 mg/kg. A study by Kiboi et al. [45] determined the ED_50_ for the sensitive parasites to be 1.67 mg/kg of LU. This therefore corresponds to an index of resistance of 55.87, confirming a high level of resistance. The fact that the ED_50_ had dropped to almost the level of the sensitive parasites by passage 17 confirms sensitivity of the parasites to LU.

For the PQ-exposed parasites, results clearly indicated that there was a fitness disadvantage on the resistant line. There was a significant difference (P < 0.05) in parasitaemia between the two lines even by day 4 pi. The PQS had a similar pattern to the LUS, with no significant difference in parasitaemia (P > 0.05) recognized despite the fact that the experiments were set at different times. This would however be expected as they were both sensitive strains, and also authenticates the reproducibility of the results observed. Observations made on PQR correspond to several previous studies which indicate that mutations associated with drug resistance confer a fitness cost. This suggests that they might disappear by reducing the volume of drug pressure, assenting that resistant forms of an organism are likely to be less fit than their wild-type sensitive strains, in the absence of selection [44,59-63]. Genetic hitch-hiking, which occurs especially among resistant parasites in the field is a detriment to them as it restricts their variability [64,65], further explaining why their frequency diminishes when active drug selection declines.

It is elucidated that the lack of expression of *pfmrp* leads to a fitness cost of the parasite in in vitro malaria culture at parasitaemias above 5%, which might be due to an impaired transport of toxic metabolites out of the parasite [65]. In the event that resistance to PQ is associated with an anomaly in *pfmrp*, then this would explain the fitness costs observed in the PQ resistant line. The mechanism of PQ resistance is however still under investigation. Despite its chemical similarity to CQ, Somé et al. [66] confirmed that the polymorphisms selected for by PQ were different from those by CQ or atovaquone (AQ), suggesting different mechanisms of resistance. PQ results confirm the theory of cost of resistance, having a five-fold faster growth rate for the wild-type parasite by D_7_ pi, and an overall resistance cost of fitness of 80.3% for the PQ-resistant line.

This study has confirmed that significant fitness costs are associated with PQ resistance and that the selective benefit acquired by becoming drug resistant is in some way biologically costly to the altered parasite [67]. It should however be noted that the strength of the fitness cost may vary between strains [36,68,69]. Furthermore, results from in vitro culture experiments or resistance mutations obtained in a laboratory in vivo system *(P. berghei)* may not be a clear representative of natural populations of *P. falciparum* [70] and therefore further studies on *P. falciparum* still ought to be performed. However, if these results are general across parasite genotypes and species, this has significance especially for mutant parasites resulting from continued population-wide exposure to drugs. This laboratory-based study provides information on the effect of resistance mutation on parasite fitness, and could certainly be extended to suggest field conditions more appropriately. For instance, by crossing genetically diverse transmissible *P. berghei* isolates in the laboratory under a range of drug treatments through successive generations [71]. An understanding of the physiological basis of fitness and genetic changes associated with resistance to a particular anti-malarial drug may lead to estimation of associated cost and potential weaknesses in resistant parasites, which can then be managed more effectively [33,72].

It was concluded that significant fitness costs are associated with PQ-resistance and this knowledge could help in establishing strategies for treatment of resistant malaria parasites. Since the work done was in vivo and thus based on observing phenotypic characteristics, a molecular approach to determine the actual genes, mutations or epigenetic factors that are involved in resistance and fitness costs due to LU and PQ still ought to be performed. Moreover, properly structured and precise laboratory studies should be performed using anti-malarial drugs such as CQ and sulfadoxine-pyrimethamine (SP) which have been withdrawn from use in Kenya for several years now. This will help establish possibility of future re-introduction of the drugs if true resistance imposes a fitness cost. In several countries such as Malawi where CQ use was suspended early enough because of widespread resistance, sensitive *P. falciparum* strains have re-emerged and are expanding [36,73]. Considering that numerous deaths from malaria are still reported in Africa, where the greatest transmission intensity occurs, this presents a ray hope in the efforts to control malaria. Future reintroduction of a drug for the management of malaria should be planned bearing in mind evolution of drug resistance, with the aim of identifying combinations that could deter the re-emergence of resistance. In malaria-endemic countries with semi-immune host populations, such as Kenya, even a partial resumption of drug sensitivity after loss to resistance, may positively impact public health.

## Abbreviations

ACT: Artemisinin-based combination therapy
ACUC: Animal Care and Use Committee
ANOVA: Analysis of variance
AQ: Atovaquone
CTMDR: Centre for Traditional Medicine and Drug Research
CQ: Chloroquine
DHA-PQ: Dihydroartemisinin-piperaquine
ED_50_: Effective dose that reduces parasitaemia by 50%
I_50_: Ratio of the ED_50_ of the resistant line to that of the parent strain
ip: Intraperitoneally
KEMRI: Kenya Medical Research Institute
LU: Lumefantrine
LUR: Lumefantrine resistant
LUS: Lumefantrine sensitive
PfMDR1: *Plasmodium falciparum* multidrug resistance transporter 1
PfCRT: *Plasmodium falciparum* chloroquine resistance transporter
PfMRP: *Plasmodium falciparum* multidrug resistance-associated protein
pi: Post-infection
PQ: Piperaquine
PQR: Piperaquine resistant
PQS: Piperaquine sensitive
PRBC: Parasitized red blood cell
WHO: World Health Organization
4DT: 4-day test
